# The effects of a leaflet-based health guide on health literacy, self-efficacy, and satisfaction among older Japanese-Brazilian adults living in Brazil: A quasi-experimental study

**DOI:** 10.1186/s12889-020-10129-1

**Published:** 2021-01-04

**Authors:** Mana Doi-Kanno, Yuka Kanoya, Emilio Hideyuki Moriguchi

**Affiliations:** 1 Nursing Course, School of Medicine, Yokohama City University, 3-9 Fukuura Kanazawa-ku, Yokohama, 236-0004 Japan; 2 Department of Internal Medicine, Federal University of Rio Grande do Sul, Av. Paulo Gama, 110 - Bairro Farroupilha, Porto Alegre, Rio Grande do Sul Brazil

**Keywords:** Health guide, Japanese-Brazilian, Older adults, Health literacy, Self-efficacy, Subjective health status

## Abstract

**Background:**

This study assessed the effects of a Japanese leaflet-based health guide for older Japanese-Brazilian adults living in Brazil, on health literacy, self-efficacy, and satisfaction with the health guide and participants’ subjective health status.

**Methods:**

The study followed a one-group pretest-posttest design and was set in the Japanese-Brazilian community in South Brazil. The 21 participants were Japanese-Brazilian individuals aged over 65 years, living in Brazil, and able to converse in Japanese. During the annual health checkup of 2016, we provided about 20 min of health guidance in Japanese using our leaflet, which included information about lifestyle-related diseases, recommended salt and sugar intake levels, and graphical charts. Participants’ health literacy (HL) was the primary outcome; self-efficacy and satisfaction with the leaflet-based health guide and participants’ subjective health status were secondary outcomes. We assessed the effect after completion of the health checkups in 2016 and 2017. Data were analyzed using repeated measures of ANOVA and the Bonferroni multiple comparison test as required.

**Results:**

There were no statistical significant differences in HL (functional HL: *p*-value = 0.22; communicative HL: *p*-value = 0.17; critical HL: *p*-value = 0.40; total HL score: *p*-value = 0.12) and self-efficacy (*p*-value = 0.28) across the three assessment points. We detected a statistical significant difference in satisfaction with the health guide, post-intervention in 2016 and 2017 (baseline score: 86.7±20.4; post-intervention score in 2016: 92.5±12.2; post-intervention score in 2017: 76.2±21.9; *p*-value of repeated ANOVA < 0.01, η_p_^2^ = 0.28; *p*-value of the multiple comparison in 2016 and 2017 = 0.01, 95% CI 4.09–28.51). However, the Bonferroni multiple comparison test did not show pairwise difference during multiple comparisons of participants’ satisfaction with their subjective health status (scores: baseline, 69.6±24.2; post-intervention in 2016, 78.5±21.1; post-intervention in 2017, 58.0±31.1; *p*-value of repeated ANOVA = 0.02, η_p_^2^ = 0.21; *p*-values of the multiple comparisons> 0.05). Scores of all outcomes, except self-efficacy, increased from baseline to post-intervention in 2016, but declined at post-intervention in 2017.

**Conclusions:**

The leaflet-based intervention appeared to have short-term effects. The findings suggest that direct intervention in older adults’ native language may improve their satisfaction when living in non-native countries.

**Trial registration:**

The UMIN-CTR unique registration ID is UMIN000032443. Retrospectively registered on May 1, 2018, at: https://upload.umin.ac.jp/cgi-open-bin/ctr_e/ctr_view.cgi?recptno=R000036999.

**Supplementary Information:**

The online version contains supplementary material available at 10.1186/s12889-020-10129-1.

## Background

Several Japanese-Brazilian families live in South Brazil, as Japanese people have been immigrating to the southern region of Brazil since 1908 [1]. Approximately 1.9 million Japanese-Brazilians currently live in Brazil [2]. The proportion of Japanese-Brazilian patients diagnosed with metabolic syndrome in Brazil (43.0–49.8%) [3], is more than that of native Japanese adults living in Japan (14.4%) [4]. This trend is more observable among older adults [3].

More than half of the Japanese-Brazilian population living in the Southern part of Brazil are not fluent in Portuguese, especially in medical terms. Reportedly, they have difficulty in understanding medical words, even if they can use Portuguese in daily life conversations [5]. A Japanese-Brazilian doctor conducts annual health checkups in several settlements where members of the Japanese-Brazilian community of South Brazil reside. About 80% of the participants attending these health checkups are older adults [5]. These present a good opportunity for older Japanese-Brazilian adults with health problems, such as obesity, hyperlipidemia, and hypertension, to obtain medical services and access them in Japanese. However, this health checkup is held once in a year. Thus, Japanese-Brazilian older adults need to manage their own health voluntarily by exchanging health information with each other and following a nutritious diet [6]. Except for this annual health checkup, there are no health resources available in Japanese, for Japanese-Brazilian adults living in Brazil. Therefore, there is a need to develop ways to provide such resources to Japanese-Brazilian adults, to help them manage their own health, in order to prevent metabolic syndrome, which puts them at greater risk of acquiring cardiovascular diseases [7, 8].

It is reported that raising individuals’ awareness of the potential factors that influence lifestyle-related diseases via a health guide may promote self-health management [9]. A better understanding of the factors and risks associated with lifestyle-related diseases, based on the use of a guide, may help individuals evaluate their health behaviors more objectively, and manage the risk factors [10]. Moreover, we expect the guide to increase individuals’ motivation to improve their health behaviors. In this study, we used a leaflet comprising graphical charts (—whereby participants can record their own data, monitor changes in their health status, and evaluate possible reasons for change in health status, which would help them better manage their health—) in the health guide session (Additional file 1).

A previous study suggested that a leaflet-based health guide may lead to more voluntary reading and use of the information therein, which might increase individuals’ awareness of the necessity of managing their own health [11]. Thus, in the present study, we aimed to develop an instructive leaflet, which includes information about lifestyle-related diseases and graphical charts for older Japanese-Brazilian adults living in Brazil.

To examine the effects of this leaflet-based health guide, we assessed participants’ health literacy (HL), self-efficacy, and satisfaction with the health guide and participants’ subjective health status, based on the assumption that our intervention would improve participants’ motivation and belief in managing their own health. Health literacy refers to the achievement of a level of knowledge, personal skills, and confidence to take action to improve personal and community health by changing one’s lifestyles and living conditions [12]. In this study, we aimed to use the health guide to improve individuals’ awareness of the potential factors, which influence lifestyle-related diseases and the necessity of managing their own health [9, 11]. Therefore, individuals’ higher level of awareness of the potential factors that influence lifestyle-related diseases and the necessity of managing their own health, i.e., higher motivation to improve their health behaviors, can be evaluated by HL; this indicates the achievement of a level of knowledge, skill, and confidence of individuals, which allows them to take action to improve their health. In addition, in order to investigate their belief in managing their own health after improving motivation, we examined general self-efficacy. Self-efficacy is defined as people’s beliefs in their capabilities to produce designated levels of performance [13]. Therefore, changes in participants’ motivation and belief can be assessed based on improvements in their HL and self-efficacy, respectively, as outcomes of our health guide.

Thus, this study aimed to assess whether a Japanese leaflet-based health guide for older Japanese-Brazilian adults living in Brazil improves their HL, self-efficacy, and satisfaction with the health guide and participants’ subjective health status. The findings of the study may provide more insights into health management approaches for people living in non-native countries.

## Methods

We used a one-group pretest-posttest design. This design used a single group of participants, and the effect of the intervention could be determined by a score comparison of the data, before and after the intervention [14].

### Patient and public involvement

Neither the patients nor the public were involved in the study design or the conduct of this study.

### Study participants

A Japanese-Brazilian doctor conducts annual health checkups in their settlements, where approximately 400 members of the Japanese-Brazilian community of South Brazil reside. This study recruited older adults who attended health checkups in 2016 at five settlements (July 23 to July 28, 2016). The inclusion criteria involved participants who (1) were Japanese-Brazilians; (2) were over 65 years of age; (3) could speak Japanese; and, (4) provided written consent to participate in the study. However, we excluded people who were not deemed fit to participate in this study, based on the advice of a doctor. Older Japanese-Brazilian adults consented to participate after understanding the outline of this study.

### Study procedure

The annual health checkups for the Japanese-Brazilian community living in South Brazil are conducted from July to August. During the health checkups, both Portuguese and Japanese languages are used; participants can select their preferred language. The health checkup includes a medical interview, electrocardiogram, measurement of body weight, body length, girth of the abdomen, blood pressure, urine examination, and laboratory tests, which are conducted in examination facilities prior to the health checkup; medical examination by a doctor; and, a 20-min health counseling session. The health counseling session is conducted at the end of health checkups, and performed by one medical staff member. The medical staff includes doctors, nurses, and medical and nursing students; students are supervised by doctors or nurses. The majority of medical staff are Japanese-Brazilians who can speak Japanese. Sometimes, Japanese medical staff who live in Japan are included as well.

**Fig. 1:**
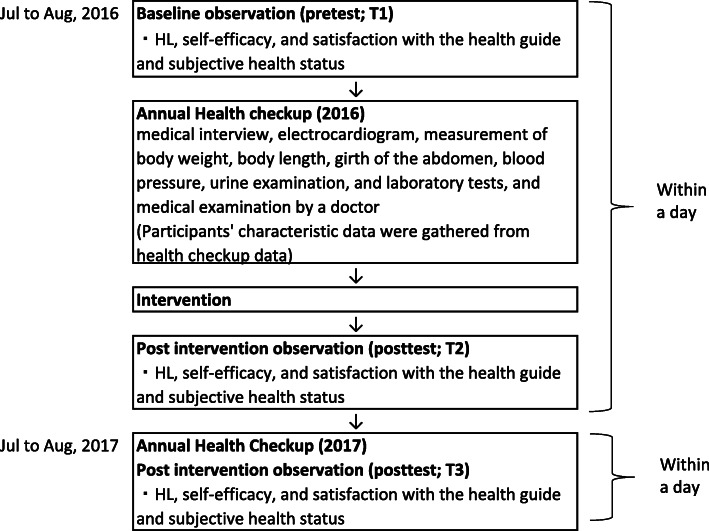
Flowchart of this study

We collected data from the participants at three time points during their annual health checkups in 2016 and 2017. We measured HL, self-efficacy, and satisfaction with the health guide and subjective health status before the health checkup in 2016 as a baseline (pretest). Participants’ characteristics such as age, health-related data (explained in detail below), and comorbidities were obtained from the 2016 health checkup data. Following that, our 20-min intervention was conducted during the 2016 health counseling sessions. HL, self-efficacy, and satisfaction with the health guide and subjective health status were also assessed after completion of the health checkups in both 2016 and 2017 (posttest assessments). Thus, our assessment time points were as follows: before the health checkup in 2016 (pretest; T1), after the health checkup in 2016 (posttest; T2), and after the health checkup in 2017 (posttest; T3). The time interval from T1 to T3 was 1 year (Fig. 1).

### Intervention

At the annual health checkup of 2016, we conducted a 20-min face-to-face health session in Japanese using our leaflet. To maintain the quality of health sessions, our interventions were conducted only by a team of Japanese researchers residing in Japan; they had practiced and prepared for the interventions in advance, in Japan. Considering participants’ deteriorating eyesight due to their age, we used a bigger font size (as large as possible) for the leaflet and provided illustrations along with tables instead of using too many words. In addition, the leaflet was printed on an A5-sized paper, which made it easy to carry, and was provided in a plastic file for safe keeping. The leaflet included (1) information about lifestyle-related diseases; (2) information about optimal levels of salt and sugar intake; and (3) graphical charts, whereby participants could record their own data from the annual health checkup. The details of the same could be found on the leaflet under the following sections: “Information about lifestyle-related diseases,” “Information about optimal levels of salt and sugar intake,” and “Graphical charts.”

During the usual annual health checkups, participants were provided with a 20-min face-to-face health counseling session. Only participants’ one-year health checkup data were recorded by the medical staff in Japanese, on A-4 size paper. Essentially, there is no difference in the kind of data that can be recorded on usual A4-size papers and our leaflet; however, our leaflet allowed annual data to be recorded for consecutive years.

### Information about lifestyle-related diseases

Information about lifestyle-related diseases provided in the leaflet was based on the literature [15–18], given that the Body Mass Index (BMI), systolic and diastolic blood pressure, and triglycerides levels of Japanese-Brazilian individuals living in Brazil are higher than those of Japanese individuals living in Japan [19]. In the leaflet, we explained that an unhealthy diet, lack of activity, intake of alcohol, smoking, and increased stress levels are associated with health-related diseases. Moreover, we introduced examples of an unhealthy lifestyle for each factor and stated that severe lifestyle-related diseases might cause cardiovascular diseases and diabetic complications, decline of physical activity, and dementia. In the last part of this section, we provide suggestions to reduce daily salt intake, smoking, alcohol consumption, and stress, and ways to improve physical activity in order to prevent lifestyle-related diseases. For the health guidance session, we required healthcare professionals to conduct sessions with participants in order to discuss measures to improve participants’ lifestyles.

### Information about optimal levels of salt and sugar intake

It is reported that Japanese-Brazilian individuals living in Brazil prefer to use salt [6] and consume sugar [5]. Thus, we showed a table that contains the amount of salt used in common Japanese foods, namely, curry and rice, Hamburg steak, dried Japanese plum, and Japanese pickles. In addition, we explained alternatives of salt, such as adequate frying. The leaflet also included the amount of sugar present in each popular juice in Brazil and the recommended daily level of sugar.

### Graphical charts

Our leaflet also included graphical charts, since these reportedly help participants to identify and evaluate their reasons for overeating and to foster health self-management [20, 21]. Participants could input their data in graphical charts recording girth of abdomen, BMI, blood pressure, triglyceride level, high-density lipoprotein (HDL) cholesterol level, low-density lipoprotein (LDL) cholesterol level, fasting blood sugar, hemoglobin A1c (HbA1c), and daily salt intake estimated from urine samples, and compare them with each cut-off point or recommended level. We expected the same from participants in the present study through their use of graphical charts reflecting upon their data from the annual health checkup, which in turn would enable them to understand their subjective health status and how it changed.

### Participants’ characteristics

Information on participants’ age, sex, primary language, BMI, blood pressure, girth of abdomen, fasting blood sugar, HbA1c, daily salt intake estimated from urine samples, triglyceride level, LDL cholesterol level, HDL cholesterol level, and comorbidities were obtained from the health checkup data.

### Outcome measures

To review HL in this study, we used the 14-item Health Literacy Scale for Japanese adults (HLS-14); total score ranges from 14 to 70, with higher scores indicating a higher level of HL [22]. HL includes functional, communicative, and critical HL. Functional literacy refers to basic skills in reading and writing; communicative HL refers to more advanced skills of extracting information, deriving meaning from different forms of communication, and applying new information to changing circumstances; critical literacy is defined as advanced skills of analyzing information critically and using this information voluntarily [22]. All subscales of HL showed good internal consistency (Cronbach’s α = 0.76–0.83). The group with the higher score of total HL more frequently thought that they could obtain medical information satisfactorily (higher score group, 67.1%; lower score group, 57.1%; *p*-value of chi-square test < 0.01) and wanted to participate in making medical decisions (higher score group, 66.3%; lower score group, 49.7%; *p*-value of chi-square test < 0.01) [22].

To measure self-efficacy, we used the Japanese version of the General Self-efficacy Scale, which is a 10-item scale. The reliability was assessed using Cronbach’s alpha test, where internal consistency value of α = 0.89. The correlation values were considered good (*r* > 0.4), with the exception of action centering (*r* = 0.15) [23]. Higher scores reflect a stronger generalized sense of self-efficacy [24].

The visual analog scale (VAS) was used to measure participants’ satisfaction with the health guide and subjective health status. Both the health guide and subjective health status were evaluated on a scale from 0 (“no satisfaction”) to 100 (“extreme satisfaction”).

We expected that the health guide would increase participants’ motivation to engage in health behaviors, and that their HL would improve. Thus, we set HL as the primary outcome. Participants’ self-efficacy and satisfaction with the health guide and their subjective health status were set as secondary outcomes.

### Statistical analysis

For HL, self-efficacy, and satisfaction with the health guide and subjective health status, we performed a repeated-measures ANOVA and multiple comparison by the Bonferroni method. The Bonferroni correction is usually used for pair-wise comparisons [25]. All data were analyzed using SPSS Statistics 25 (IBM Corp., Armonk, NY, USA) and the significance level was set at α = 0.05.

### Ethics

This study was approved by the Institutional Review Board of the Yokohama City University (no. B160602001), and Federal University of Rio Grande do Sul (Universidade Federal do Rio Grande do Sul PROEXT (Pró-Reitoria de Extensão) Projeto # [28405]). We guaranteed the protection of personal information and encouraged voluntary participation. Written informed consent was obtained from all participants who were enrolled at the baseline. We collected data, which related to this study, anonymously. The data was stored and managed in both Brazil and Japan by the authors. Only researchers could access the data.

## Results

In this study, 47 participants consented to participate, and data from 21 (mean age: 75.3 years; standard deviation [SD]: 5.6 years; males: 54.1%; females: 45.9%) participants were finally analyzed. There were no significant differences in the characteristics of participants whose data were analyzed and those who were lost to follow-up (*p*> 0.05). The characteristics of the participants are presented in Table 1.

**Table 1:** Participants’ characteristics *n*=21

	mean±SD
Age (years)	75.3±5.6
Sex (male, %)	54.1
Language (%)	
Japanese only	14.3
Japanese and Portuguese	85.7
BMI (kg/m^2^)	24.3± 2.5
Systolic blood pressure (mmHg)	149.1±18.9
Diastolic blood pressure (mmHg)	78.0±10.1
Girth of abdomen (cm) (*n*=20)	89.9± 8.2
Fasting blood sugar (mg/dL) (*n*=12)	131.6±24.6
HbA1c (%) (*n*=20)	6.1±0.7
Salt intake (g) (*n*=10)	9.9±1.9
Triglycerides (mg/dL) (*n*=20)	150.9±92.8
HDL-C (mg/dL) (*n*=20)	58.4±14.7
LDL-C (mg/dL) (*n*=19)	87.0±26.5
Diabetes (%)	23.8
Hypertension (%)	28.6
Hyperlipidemia (%)	23.8

**Fig. 2:**
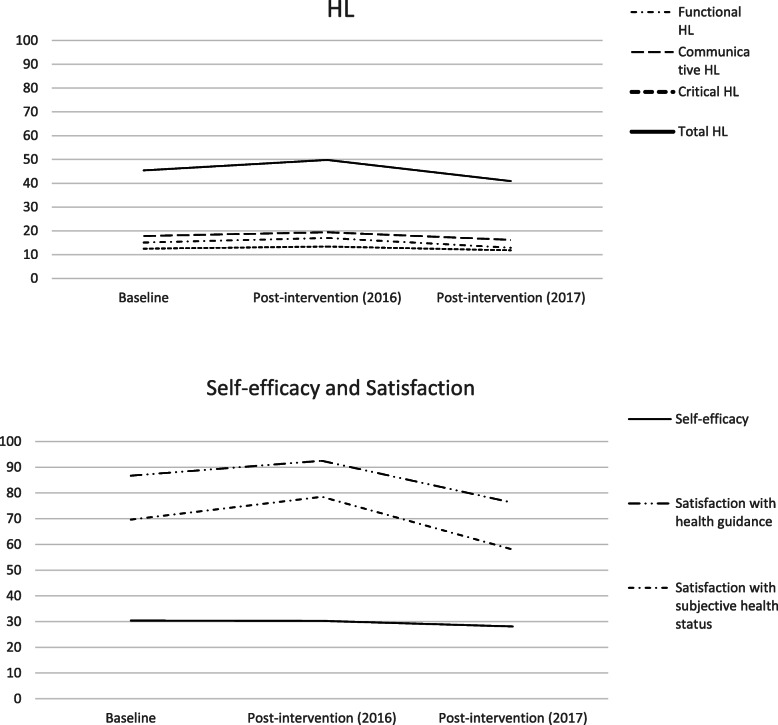
Score trends for participants’ HL, self-efficacy, and satisfaction with the health guide and subjective health status. Abbreviations: HL, health literacy

Table 2 shows scores of HL, self-efficacy, and satisfaction with the health guide and participants’ subjective health status. There were no statistical significant differences in participants’ HL (functional HL: 15.1±5.7 at baseline; 17.0±6.6 at post-intervention in 2016; 12.9±5.6 at post-intervention in 2017; *p*-value=0.22; communicative HL: 17.8±6.5 at baseline; 19.4±6.3 at post-intervention in 2016; 16.2±6.0 at post-intervention in 2017; *p*-value=0.17; critical HL: 12.5±4.9 at baseline; 13.4±5.1 at post-intervention in 2016; 11.8±4.0 at post-intervention in 2017; *p*-value=0.40; total scores of HL: 45.4±13.1 at baseline; 49.8±12.8 at post-intervention in 2016; 40.9±12.0 at post-intervention in 2017; *p*-value=0.12) and self-efficacy (30.4±7.3 at baseline; 30.3±7.4 at post-intervention in 2016; 28.1±7.1 at post-intervention in 2017; *p*-value=0.28). Regarding participants’ satisfaction with the health guide, we detected a statistical significant difference at post-intervention in 2016 and 2017 (baseline score: 86.7±20.4; post-intervention score in 2016: 92.5±12.2; post-intervention score in 2017: 76.2±21.9; *p*-value of repeated-measures ANOVA < 0.01, η_p_^2^ = 0.28; *p*-value of the multiple comparison in 2016 and 2017 = 0.01, 95%CI 4.09–28.51). In the multiple comparisons of participants’ satisfaction with their subjective health status, the Bonferroni multiple comparison did not show pairwise difference (baseline score: 69.6±24.2; post-intervention score in 2016: 78.5±21.1; post-intervention score in 2017: 58.0±31.1; *p*-value of repeated-measures ANOVA = 0.02, η_p_^2^ = 0.21; *p*-values of the multiple comparisons > 0.05). The scores of all outcomes, except self-efficacy, increased from baseline to post-intervention in 2016, but declined at post-intervention in 2017 (Fig. 2).

**Table 2:** Scores of participants’ HL, self-efficacy, satisfaction with the health guide and subjective health status *n*=21

		mean±SD			*p*-value^a^	Multiple comparison mean difference and *p*-value^b^		
		2016		2017		T1 vs. T2	T1 vs. T3	T2 vs. T3
		Baseline T1	Post-intervention (2016): T2	Post-intervention (2017): T3				
HL (*n*=17)	Functional HL	15.1±5.7	17.0±6.6	12.9±5.6	0.22			
	Communicative HL	17.8±6.5	19.4±6.3	16.2±6.0	0.17			
	Critical HL	12.5±4.9	13.4±5.1	11.8±4.0	0.40			
	Total HL	45.4±13.1	49.8±12.8	40.9±12.0	0.12			
Self-efficacy (*n*=18)		30.4±7.3	30.3±7.4	28.1±7.1	0.28			
Satisfaction	with health guide (*n*=20)	86.7±20.4	92.5±12.2	76.2±21.9	< 0.01*	5.8 (T2-T1) *p*=0.26	−10.5 (T3-T1) *p*=0.14	−16.3 (T3-T2) *p*=0.01*
	with subjective health status (*n*=19)	69.6±24.2	78.5±21.1	58.0±31.1	0.02*	8.9 (T2-T1) *p*=0.33	−11.6 (T3-T1) *p*=0.25	−20.5 (T3-T2) *p*=0.06

^a^repeated-measures ANOVA ^b^Multiple comparison by Bonferroni procedure (adjusted *p*-value)

^*^p< 0.05

## Discussion

The leaflet-based health guide improved participants’ HL and satisfaction with the health guide and their subjective health status at post-intervention; their scores declined after a year. However, we detected a significant difference in satisfaction between the health guide post-intervention in 2016 and that in 2017; there was no statistical difference in other aspects of comparison.

We expected that the health guide, which provides information about lifestyle-related diseases, would help participants objectively evaluate their health behaviors and increase their motivation to engage in healthy behaviors [10], which in turn would improve their HL. In this study, we found that participants’ HL scores were improved at post-intervention in 2016, although this was not statistically significant. We also expected improvement in self-efficacy, because a previous study reported an association between HL and self-efficacy [26]. However, scores of general self-efficacy did not improve at post-intervention in 2016. The short-term improvements observed after using the health guide could be explained by the fact that only motivation improved, while there was no increase in self-efficacy, thus indicating no improvement in their belief in health behavior change.

In addition, we found an improvement in participants’ satisfaction with the leaflet-based health guide and their subjective health status at post-intervention in 2016. Participants’ satisfaction likely improved due to three reasons. First, we showed the amount of salt and sugar present in common foods, along with tables and figures to provide a better understanding of how participants could modify their dietary habits (e.g., consuming only half a bottle of juice). Second, we used a larger font size (mainly used font size 16 for an A5-sized leaflet) and colored illustrations in our leaflet. Most older adults experience changes in visual acuity and accommodation due to presbyopia [27]. Thus, the larger font size made it easier for participants to read the information. Third, a visual representation of their data using graphical charts improved participants’ satisfaction with their subjective health status. Prior to this study, participants were provided only with their one-year health checkup data, recorded only by the medical staff. However, in this study, participants could record their health status in their own leaflet, which enabled them to understand and assess their changing health status over the years. Therefore, the new ideas presented in the leaflet-based health guide presumably improved participants’ satisfaction with both subjective health status and the health guide.

However, the overall scores for each outcome declined after a year; especially, the scores of satisfaction with the health guide decreased significantly. Thus, our leaflet-based health guide appears to have short-term effects. This is because the intervention in 2016 was implemented in Brazil by a team of Japanese researchers residing in Japan, who provided face-to-face health guidance comprising our leaflet to all participants. In 2017, participants received the usual health guide. These results suggest that the face-to-face intervention by Japanese researchers improved participants’ satisfaction with the health guide. Moreover, although the leaflet was developed to support older adults living in non-native countries, such as Japanese-Brazilian people living in Brazil, where it is difficult to provide long-term face-to-face support, the effects of the leaflet-based health guide had diminished. A face-to-face approach might be effective in this regard, because older adults need an attentive approach [27]. In future, it is recommended that a tele-health guide be provided to older Japanese-Brazilian people living in South Brazil to enable long-distance face-to-face support.

### Strengths and limitations

The strength of our study is that we provided a face-to-face intervention for older adults living in Brazil and followed it up the next year. Although the leaflet-based guide was deemed to have only short-term effects, our findings suggest that consideration of a tele-health guide for older adults living in non-native countries, may enable long-term follow-up.

The small sample size due to declined follow-up rates is a limitation of this study, which possibly led to the large post-intervention difference observed. These declined follow-up rates could be due to the fact that we were unable to send the two Japanese research assistants to Brazil because of the political situation of Brazil, in 2017. Medical staff of southern Brazil had to carry out both the health checkups and collect data for our study, in 2017, with limited human resources. Because of this selection bias, there is a possibility that this influenced our results; it could be possible that the majority of our study participants were indeed highly interested in their health. In addition, study participants were selected from a small region of South Brazil, which limits the generalizability of the study findings. Moreover, we cannot control for the possibility that our findings might be influenced by phenomena, such as weather and political events in Brazil, in 2016 and 2017, because we used a one-group pretest-posttest design. Therefore, a large-scale study with adequate human resources must be conducted. Future studies should examine the long-term effects of our leaflet-based health guide with a larger sample and develop a tele-health guide for older adults living in non-native countries.

## Conclusion

The leaflet-based health guide improved participants’ scores of HL, and satisfaction with the health guide and their subjective health status from baseline to post-intervention in 2016, but declined at post-intervention in 2017. While our leaflet-based health guide appeared to have only short-term effects, our findings suggest that providing a direct intervention, such as a face-to-face approach, may improve older adults’ functional, communicative, and critical HL, as well as their satisfaction with the health guide and their subjective health status, especially for those living in non-native countries.

## Supplementary Information

**Additional file 1.** One page of the graphical chart (the English version). This is one page of the graphical chart. This page is for the measurements girth of abdomen and BMI.

## Data Availability

The data set is not available for access and/or publication as the ethics approval body does not allow it.

## Abbreviations

BMI: Body mass index
HbA1c: Hemoglobin A1c
HDL: High-density lipoprotein
HL: Health literacy
HLS-14: 14-item Health Literacy Scale for Japanese adults
LDL: Low-density lipoprotein
VAS: Visual analog scale
