# Supplementary material for: The effects of a leaflet-based health guide on health literacy, self-efficacy, and satisfaction among older Japanese-Brazilian adults living in Brazil: A quasi-experimental study
Source: BMC Public Health. 2021 Jan 4;21:10. doi: 10.1186/s12889-020-10129-1 (PMC7784267; doi:10.1186/s12889-020-10129-1)
Supplement: Supplementary file 1 — Additional file 1. One page of the graphical chart (the English version). This is one page of the graphical chart. This page is for the measurements girth of abdomen and BMI. [file 12889_2020_10129_MOESM1_ESM.docx]

**Girth of abdomen**

|  |  |  |  |
| --- | --- | --- | --- |
|  |  |  |  |
|  |  |  |  |
|  |  |  |  |
|  |  |  |  |
|  |  |  |  |
|  |  |  |  |

92

For female

90

88

86

For male

84

82

80

78

2016 2017 2018

**BMI (Body Mass Index)**

BMI

Weight (kg)

Height (m)

Height (m)

　　　　　　÷　　　　　　　÷　　　　　　　＝

|  |  |  |  |
| --- | --- | --- | --- |
|  |  |  |  |
|  |  |  |  |
|  |  |  |  |
|  |  |  |  |
|  |  |  |  |

30

28

26

24

22

20

18

2016 2017 2018
